# Chemical Composition, Antibacterial and Radical Scavenging Activity of Essential Oils from *Satureja macrantha* C.A.Mey. at Different Growth Stages

**DOI:** 10.3390/foods9040494

**Published:** 2020-04-14

**Authors:** Behzad Nezhadasad Aghbash, Mohammad Pouresmaeil, Gholamreza Dehghan, Mohsen Sabzi Nojadeh, Haedeh Mobaiyen, Filippo Maggi

**Affiliations:** 1 Department of Biology, Faculty of Basic Sciences, Shahed University, Tehran 3319118651, Iran; Behzad123asad@gmail.com; 2 Department of Plant Sciences, Faculty of Natural Sciences, University of Tabriz, Tabriz 5166616471, Iran; 3 Faculty of Natural Sciences, University of Tabriz, Tabriz 5166616471, Iran; dehghan2001d@yahoo.com; 4 Ahar Faculty of Agriculture and Natural Resources, University of Tabriz, Ahar 5354854517, Iran; m.sabzinojedeh@gmail.com; 5 Department of Microbiology, Tabriz Branch, Islamic Azad University, Tabriz 5166616471, Iran; drmobaiyen@gmail.com; 6 School of Pharmacy, University of Camerino, 62032 Camerino, Italy

**Keywords:** *Satureja macrantha*, essential oil, food preservative, antibacterial activity, antioxidant activity

## Abstract

Essential oils (EOs) from medicinal and aromatic plants are interesting products to be used as natural food preservatives. The EOs from the genus *Satureja* are reported to inhibit foodborne pathogens being worthy of use as food preservatives. *Satureja macrantha* is found in Western and Northwest Iran and commonly used as a food flavoring agent and for the treatment of urinary diseases. The objective of the present study was to identify the chemical composition of *S. macrantha* EOs at different growth stages (vegetative, flowering and fruiting stages) and to evaluate their biological activities. Chemical compositions were analyzed using GC-FID and GC-MS. The antibacterial activity was evaluated using the broth microdilution method against the foodborne pathogenic bacteria *Staphylococcus aureus* (ATCC23922), *Enterococcus faecalis* (ATCC29212) (Gram-positive), *Enterobacter aerogenes* (ATCC13046) and *Escherichia coli*. The antioxidant activity was estimated using the DPPH, ABTS and reducing power assays. The yields of *S. macrantha* EOs were in the range of 1.4–1.8%, thus scalable for the manufacture of food preservatives on an industrial level. The main compounds of EOs were carvacrol (42.7–48.2%), thymol (0.2–16.5%), *p*-cymene (10.1–14.7%) and γ-terpinene (7.9–9.1%) in all phenological stages examined. The strongest antibacterial activity (MICs = 5–20 µg/mL) of the EOs was recorded in samples obtained during the flowering stage where carvacrol (42.7%) and thymol (16.5) were present both at high percentages. On the other hand, the antioxidant activity was found to be slightly higher in the other stages. As the EO obtained at flowering showed the best inhibitory properties against foodborne pathogenic bacteria, it is suggested that plants at this stage can be selected as main sources of food preservative agents.

## 1. Introduction

In recent years, the impact of synthetic antibiotics and antioxidants used as food preservatives on human health and the environment is matter of concern [1]. Moreover, the increase of bacterial antibiotic resistance pushed the food industry to look to alternative strategies relying on natural sources such as plant essential oils (EOs) and extracts [2,3]. EOs are natural mixtures of volatile and hydrophobic compounds produced by medicinal and aromatic plants (MAPs) and obtained by the classical process of steam distillation, or cold pressing in the case of citrus fruits. These mixtures have a strong odor and are characterized by several classes of chemical compounds such as monoterpenoids, sesquiterpenoids and phenylpropanoids [4]. Recently, EOs have been extensively considered for their useful biological functions, particularly antimicrobial and antioxidant activity. Due to safety concerns related to the use of synthetic compounds, EOs have received more attention as a flavoring and a natural preservative in the food industry. Their application as food preservatives is related to their documented antimicrobial and antioxidant activities. Some compounds occurring in EOs target the bacterial cell membrane, altering its permeability and causing an increase of fluidity, disruption of the proton motive force and interference with the cellular energy generation system [5]. Moreover, the possibility to inhibit the bacterial quorum sensing is an interesting hallmark of plant EOs [6]. The antioxidant activity of EOs is closely linked with the reactive oxygen species scavenging activity and inhibition of lipid peroxidation displayed by some constituents. Both EO antimicrobial and antioxidant activities may help to increase the shelf life of foods [5]. Thus, earlier studies explored the potential of EOs as food preservatives focusing on their inhibitory properties against foodborne pathogens and antioxidant capacities.

*Satureja macrantha* is member of the genus *Satureja* (*Lamiaceae*), containing 54 species distributed in Asia, the Mediterranean area and America. Fourteen species of this genus are found in Iran and distributed in the mountainous areas [7]. This genus includes aromatic shrubs endowed with glandular hairs covering leaves and flowers and exuding an appreciable EO [8,9]. It is interesting to note that several members of this genus are reported to inhibit foodborne pathogens being worthy of use as food preservatives [10,11].

*Satureja macrantha* C.A.Mey. is a small shrub, 30 to 50 cm tall, with stems covered by trichomes and linear leaves. This species is distributed in Western and Northwestern Iran [12]. In Iranian folk medicine, it is used for the treatment of several diseases including diarrhea, wounds, gastroenteritis and upper respiratory and urinary tract infections [13]. The herbaceous parts and green leaves are also used as food-flavoring agents [14].

The EOs of the *Satureja* species such as *S. macrantha* [14,15,16], *S. montana* L., *S*. *subspicata* Bartl. ex Vis. [17], *S. mutica* Fisch. and C.A.Mey., *S. intermedia* C.A.Mey [14] and *S. sahendica* Bornm. [18] are characterized by high amounts of the phenolic monoterpenes thymol and carvacrol. These compounds have been accepted by the European Commission and the United States Food and Drug Administration (FDA) for use as flavoring agents in foods and are classified as generally recognized as safe (GRAS) [19]. Thus, many investigations have been carried out on the antimicrobial and antioxidant properties of the genus *Satureja*. Azaz et al. (2005) [15] tested the antibacterial properties of *S. hortensis* L., *S. cuneifolia* Ten., *S. thymbra* L., *S. aintabensis* P.H. Davis and *S. macrantha* EOs on several bacterial and fungal strains, indicating significant antibacterial and antifungal potential. The EOs of *S. bachtiarica* Bunge demonstrated antibacterial effects against *Staphylococcus aureus* isolated from milk [20]. Another investigation on the effect of this EO on the isolates of *Helicobacter pylori* showed an inhibitory activity on this bacteria strain [21]. *Satureja calamintha* (L.) Scheele EO has been investigated for its inhibitory potential on a wide spectrum of foodborne pathogens such as *S. aureus*, *Listeria monocytogenes*, *Enterococcus faecium*, *Yersinia enterocolitica*, *Salmonella senftenberg*, *Escherichia coli* and *Bacillus subtilis* [22].

The antioxidant activity of the EOs from the genus *Satureja* has also been reported in terms of its potential use in the food industry. The antioxidant activity of the *S. macrantha* EO has been reported by Ghorbanpour et al. [23]. Application of the *S. thymbra* EO in gilthead seabream fillets’ edible coating extended the fish shelf life by up to 35% [24]. The EO of *S. cilicica* P.H. Davis showed antioxidant activity in butter [25]. The *S. montana* and *S. subspicata* Bartl. ex Vis. EOs have shown significant radical scavenging activities [17]. The *S. avromanica* Maroofi EO was found to be active against *S. aureus*, *B. cereus* and *B. pumilus* [26]. The production, composition and yield of EOs in MAPs can be affected by several factors such as harvesting times and growth stages [27,28]. To the best of our knowledge, there are no studies on the investigation of the phytochemical profile of *S. macrantha* EOs and their antibacterial and antioxidant activity at different phenological stages. Therefore, this study is aimed at: (1) analyzing the chemical composition of *S. macrantha* during three stages, namely vegetative, flowering and fruiting, and (2) evaluating the EOs antioxidant capacity and the inhibitory properties on several important bacterial strains related to food spoilage.

## 2. Materials and Methods

### 2.1. Plant Sampling

Aerial parts of *S. macrantha* were collected at vegetative (May 2017), flowering (July 2017) and fruiting (September 2017) stages around Marand city, East Azarbaijan province, Iran (38°44′44.88″ N, 45°36′35.73″ E, 1290 m a.s.l.). The plants were identified and authenticated by Amir Hossein Talebpour and voucher specimens (SM1263-1265) were deposited at East Azerbaijan Research and Education Centre for Agricultural and Natural Resources. Annual rainfall, relative humidity and mean temperature of the region where the plants grow, were 386.7 mm, 51% and 12.2 °C, respectively. Once collected, the plant material was air-dried for one week and ground before isolation of EOs.

### 2.2. Isolation of the EOs

Two hundred fifty grams of the plant samples were subjected to hydro-distillation using a Clevenger-type apparatus for 3 h [29]. This was repeated three times for each sample obtained at each stage. The EOs’ yield was calculated based on a dry weight according to the following formula: EO yield = [amount (g) of obtained EO/amount of dry material (g)] × 100. The obtained EOs were dried over anhydrous Na_2_SO_4_ (Merck, Darmstadt, Germany) and kept at 4 °C until analysis.

### 2.3. Gas Chromatography (GC-FID) and Gas Chromatography-Mass Spectrometry (GC-MS)

Analyses of EOs were performed on a gas chromatograph (Agilent Technologies, Agilent 6890N, Santa Clara, CA, USA) connected to a mass detector (Agilent Technologies, Agilent 5973N, Santa Clara, USA) and a flame ionization detector (FID) using the same operative conditions. An HP-5MS (5% phenylmethylpolysiloxane) capillary column (30 m × 0.25 mm i.d., film thickness 0.25 µm) was used as a stationary phase. The carrier gas was helium at a flow rate of 1.5 mL/min and with a split ratio of 1:50. Oven temperature was set up to 60 °C and kept at this temperature for 4 min and then increased up to 250 °C with a rate of 5 °C /min. The injector and detector temperatures were 200 °C and 250 °C, respectively. Spectra were acquired in the mass range of 50–500 *m/z* in electron impact mode using an ionization voltage of 70 eV. The EOs were diluted 1:10 in dichloromethane and 0.1 µL were injected into the GC systems. Moreover, a mixture of *n*-alkanes (C_8_–C_20_) (Merck, Darmstadt, Germany) was injected into the system with the above-mentioned conditions to determine the retention index (RI). The compounds were identified through a comparison of the obtained mass spectra with those saved in the NIST (NIST05a.L) and Wiley (wiley7n.L) computer libraries and were confirmed by correspondence with RI reported in the literature [30]. The relative percentage of each compound was calculated using GC peak areas without any correction factors.

### 2.4. Antibacterial Screening

#### 2.4.1. Bacterial Strains

Four referencial and most-frequent foodborne pathogenic bacteria strains including Gram-positive *Staphylococcus aureus* (ATCC23922), *Enterococcus faecalis* (ATCC29212), and Gram-negative *Enterobacter aerogenes* (ATCC13046) (also known as *Klebsiella aerogenes*) and *Escherichia coli* (ATCC25922) were selected to examine the antibacterial activity of the EOs. These were provided by Dr. Haedeh Mobaiyen from the Department of Microbiology, Azad University of Tabriz, Iran. 

#### 2.4.2. Minimum Inhibitory Concentration (MIC) 

Antibacterial activity of *S. macrantha* EOs at different growth stages was evaluated using the broth micro-dilution method based on the estimation of the minimum inhibitory concentration (MIC) [31]. The stock solutions of the EOs were prepared in dimethylsulphoxide (DMSO) (Sigma Aldrich, Darmstadt, Germany) and two-fold serial dilutions were inserted in 96-well microtitre plates. The bacterial suspensions in Muller Hinton broth (Merck, Darmstadt, Germany) were standardized to 106 CFU/µL and then added to microtiter plates with EO concentrations from 2.5 to 160 µg/L. Gentamicin (Sigma Aldrich, Darmstadt, Germany) and DMSO were used as positive and negative controls, respectively. The microtiter plates were incubated at 37 ± 2 °C for 72 h. The first plate that contained no visibility grown bacteria was considered as the MIC. 

#### 2.4.3. Minimum Bactericidal Concentration (MBC)

Ten µL of the MIC-determined microtiter plates were placed on Muller Hinton agar plates and then incubated at 37 ± 2 °C for 48 h. The lowest concentration which indicated no bacterial growth was considered as the minimum bactericidal concentration (MBC). 

### 2.5. Antioxidant Activity 

The antioxidant activity of the EOs was evaluated based on the ability of the EOs to scavenge the free radical diphenyl-1-picrylhydrazyl (DPPH) by spectrophotometric method [32]. Briefly, 50 µL of the diluted samples with a range of 10–100 µg/mL (prepared in methanol solvent) were mixed with 5 mL of DPPH (0.004%) (Sigma Aldrich, Darmstadt, Germany). After 30 min, the absorbance of the samples was recorded at 517 nm against the control using a UV-visible spectrophotometer (Analytik Jena, SPEKOL-1500, Munich, Germany). The inhibition percentage was calculated according to the Equation (1):

Inhibition percent = (Abs of control − Abs of sample)/(Abs of control) × 100(1)

The IC_50_ (µg/mL) values were calculated as the concentration of each sample, which neutralizes 50% of DPPH free radicals. Under the same conditions, the inhibition percentage and IC_50_ of butylated hydroxytoluene (BHT) (Sigma Aldrich, Darmstadt, Germany) as a positive control was calculated. 

For the assessment of the antioxidant activity of EOs based on the ABTS method [33], 7 mM ABTS·^+^ (Sigma Aldrich, Darmstadt, Germany) was prepared and mixed with 2.45 mM ammonium persulfate (Sigma Aldrich, Darmstadt, Germany) and kept under darkness for 16 h. Afterwards, the ABTS·^+^ solution was diluted 50-fold with ethanol (Sigma Aldrich, Darmstadt, Germany) and one mL of ABTS·^+^ was mixed with one mL of the various concentrations of EOs (50, 100, 150 and 200 µg/mL in ethanol). The absorbance of the samples was read at 734 nm. The inhibition percentage was calculated using a similar formula as that of the DPPH method and EC_50_ values were calculated.

For the assessment of the EOs reducing power, one mL of each EOs concentration (10–100 µg/mL prepared in methanol) was mixed with 2.5 mL of phosphate buffer (0.2 M, pH 6.6) (Sigma Aldrich, Darmstadt, Germany) and 2.5 mL of potassium ferricyanide (1%) (Merck, Darmstadt, Germany), and then was incubated in a water bath at 50 °C for 20 min. After incubation, 2.5 mL of trichloroacetic acid (10%) (Merck, Darmstadt, Germany) were added and centrifuged at 3000 g for 10 min. Next, 2.5 mL of the supernatant were mixed with 2.5 mL of distilled water and 0.5 mL ferric chloride (0.1%) (Merck, Darmstadt, Germany) solution. The absorbance of the samples was recorded at 700 nm using a UV-visible spectrophotometer (Analytik jena, SPEKOL, 1500, Munich, Germany) [33].

### 2.6. Data Analysis 

All analyses were performed in three replications and presented as mean ± standard deviation (SD). Statistical analysis was carried out using SPSS software ver. 22. Differences were tested according to Duncan’s multiple tests in the general linear model (GLM) at *p* ≤ 0.05. 

## 3. Results and Discussions 

### 3.1. Essential Oil Analysis

As shown in Table 1, a variability in yields and essential oil compositions were observed for *S. macrantha* samples obtained at different phenological stages. The highest EO yield was recorded at the flowering stage (1.8%) while the lowest content was observed at the fruiting stage (1.4%). The yield values observed in the present study were comparable to those previously reported by Sefidkon and Jamzad [14] and Gohari et al. [16], who found values ranging from 0.30% to 1.48%. The highest EO yield at the flowering stage is a usual phenomenon observed in many MAPs, in particular with those belonging to the *Lamiaceae* family [34,35]. This can be explained as an outcome of enhanced photosynthetic activity during flowering which boosts the growth of glandular trichomes [36] and biosynthesis of secondary metabolites compared to vegetative and post-flowering stages [35].

**Figure 1:**
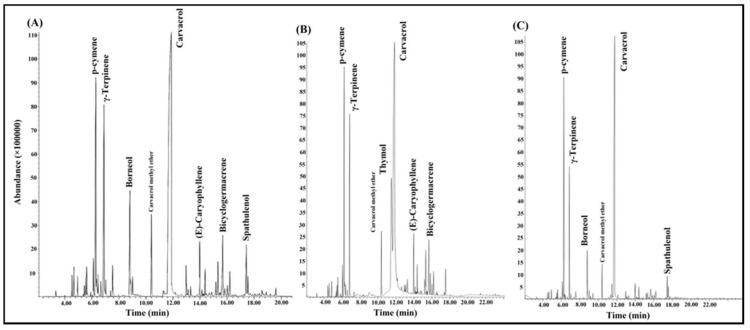
GC-MS chromatograms of the essential oils of *Satureja macrantha* at vegetative (**A**), flowering (**B**) and fruiting (**C**) stages.

The GC/MS analysis allowed us to identify a total of 42 compounds in the EOs obtained at the vegetative, flowering and fruiting stages, representing 96.7, 99.1 and 92.5% of the total composition, respectively (Figure 1, Table 1). *p*-Cymene, γ-terpinene and carvacrol were the major compounds at all phenological stages. The main compounds at the vegetative stage were: carvacrol (48.2%), *p*-cymene (10.1%) and γ-terpinene (9.1%); at the flowering stage: carvacrol (42.8%), thymol (16.5%), *p*-cymene (11.1%) and γ-terpinene (7.9%); at the fruiting stage: carvacrol (45.6%), *p*-cymene (14.7%) and γ-terpinene (8.0%). *S. macrantha* EOs were characterized by a rich presence of oxygenated monoterpenes (from 55.6% at fruiting to 63% at flowering) followed by monoterpene hydrocarbons (from 23.4% at flowering to 27% at fruiting). Furthermore, a significant amount of sesquiterpene hydrocarbons (11.4%) were found at the flowering stage. Our results were in agreement with previous research where carvacrol, *p*-cymene, γ-terpinene and thymol were found as the marker volatiles of *S. macrantha* [14,15,16]. The thymol content was higher at the flowering stage compared to the other two stages. During the vegetative stage, carvacrol and γ-terpinene achieved the highest amount while *p*-cymene reached its highest level at the fruiting stage. As thymol and carvacrol are important bioactive compounds supporting applications in pharmaceutical and food industries [37,38,39,40,41], the flowering stage of *S. macrantha* can be the best one to obtain a high quality EO. Previous studies indicated that thymol and carvacrol can be found in high amounts at the flowering stage in other *Lamiaceae* and *Apiaceae* species [37,42,43]. Chemical composition depends on pedoclimatic factors (microclimate within the soil), harvesting time and developmental stage of plants [42,44]. In this respect, some studies conducted on *Thymus vulgaris* L., *Anthemis wiedemanniana* Fisch. and C.A.Mey., *Daucus sahariensis* Murb. and *Echinophora tenuifolia* (Guss.) Tutin revealed the influence of growth stages on the quali-quantitative composition of EOs [43,45,46,47].

### 3.2. Antibacterial Activity of the Satureja Macrantha EOs

The antibacterial potential of the EOs from *S. macrantha* samples collected at different growth stages was evaluated using determination of MIC and MBC against four important bacterial strains (Table 2). The results showed both variability in the antibacterial activity of EOs collected at different growth stages and bacterial susceptibility. Among the bacterial strains, *S. aureus* was the most sensitive strain to the EOs (MIC of 5–10 µg/mL and MBC of 7.5–10 µg/mL) while *E. faecalis* was the most resistant one (MIC and MBC in the range of 20–60 µg/mL). Moderate sensitivity to EOs was exhibited by *E. aerogenes* (MIC and MBC in the range of 7.5–40 µg/mL) and *E. coli* (MIC and MBC in the range of 10–20 µg/mL). In this study, the EO obtained at the flowering stage exhibited the greatest activity (with MIC in the range of 5–20 µg/mL and MBC in the range of 7.5–20 µg/mL) while the one obtained at the vegetative stage showed the lowest antibacterial properties (MIC and MBC in the range of 10–80 µg/mL).

There are no reports on the antibacterial activity of *S. macrantha* EOs depending on different phenological stages. The antibacterial activity of this plant has been previously tested on several strains such as *E. coli*, *S. aureus*, *P. aeruginosa* and *K. aerogenes*, and MIC values were reported in the range of 31.25–125 µg/mL [15]. Furthermore, the antimicrobial activity of the EOs from several *Satureja* species such as *S. montana*, *S. subispicata* and *S. avromanica*, was previously investigated on *Brochothrix thermosphacta*, *E. coli*, *Listeria innocua*, *Pseudomonas putida*, *Salmonella typhimurium*, *B. subtilis*, *S. aureus*, *E. faecalis*, *S. epidermidis*, *Klebsiella pneumoniae*, *P. aeruginosa*, *B. cereus*, *B. pumilus*, *Saccharomyces cerevisiae* and *Candida albicans*, reporting MIC values ranging from 0.78 to 6.25 µg/mL [26,48]. The EOs’ antibacterial properties are strictly related to the chemical composition which may in turn be influenced by various factors, in particular during the phenological stages [49,50]. In this study, the EO from the flowering aerial parts exhibited the highest antibacterial activity which was related to the abundance of monoterpene phenols (thymol and carvacrol). However, other compounds with a low percentage such as *p*-cymene and γ-terpinene can play an important role in the EOs’ bioactivity. Moreover, the other EO samples obtained at the vegetative and fruiting stages showed a good antibacterial activity which in turn can be related to the presence of the same compounds, namely thymol, carvacrol and *p*-cymene. The highest antibacterial activity displayed by the EO obtained at flowering may be related to the possible additive/synergistic effects of thymol and carvacrol that are found in higher amount in this sample. Thymol and carvacrol are considered effective natural antimicrobial agents. They are capable of aligning with fatty acid chains of lipid bilayers by interacting with transmembrane proteins and forming channels through the membrane leading to the increase of membrane fluidity and alteration of proton motive force and cell permeability. They are also capable of damaging the outer membrane of Gram-negative bacteria [51] and interfering with cellular energy (ATP) generation systems [52]. On the other hand, *p*-cymene and γ-terpinene are biosynthetically related to thymol and carvacrol [53,54,55] and their presence may lead to synergistic effects in the bacterial cell [56].

Earlier studies have demonstrated the influence of the phenological stages on the overall antibacterial nature of EOs. Notably, our results are in agreement with the findings of Mirjana et al. [57] who investigated the antibacterial activity of *S. cuneifolia* EOs and reported that the bacterial strains were more sensitive to the EOs obtained during the flowering stage.

Despite EOs have been suggested as natural agents in food preservation, there are some challenges to be faced in developing this kind of products on an industrial level. One of the limitations is that high concentrations of EOs are needed to achieve sufficient antimicrobial activity. This may cause changes in the quality and taste of the foods. Interaction of the EO compounds with food matrix components is another limitation. It has been reported that EO constituents can be impaired by food matrix components such as proteins, fat and starch and this requires higher concentrations of use [19]. For example, a study showed that the concentration of Cilantro EO needed to achieve a significant antimicrobial activity was 0.018%, while in the ham model, even the 6% concentration did not show antimicrobial activity [58]. On the other hand, the EOs can cause several side effects on humans such as poisoning, skin sensitization and allergy as well as alteration of food organoleptic properties. For this reason, before applying for an application of EOs as food preservatives, an appropriate encapsulation strategy together with a comprehensive evaluation of its organoleptic and toxicity impacts should be addressed [19,59,60].

### 3.3. Antioxidant Activity 

The antioxidant activity of the EOs from *S. macrantha* obtained at different growth stages was determined using DPPH, ABTS and reducing power assays (Table 3). The activity of the synthetic antioxidant BHT was determined for comparative purposes. EOs are complex mixtures including a wide range of chemical compounds with different behaviors, functional groups and polarity. Therefore, the employment of multiple methods to evaluate the antioxidant activity can give increasing information on their antioxidant properties [61,62]. The different behavior of EOs in various methods can represent the oxidant/antioxidant models and or lipophilic/hydrophilic properties of the EOs’ components [36].

The DPPH assay was used to estimate the radical scavenging activity of EOs [63]. In this assay, a significant (*p* ≤ 0.05) variation in the antioxidant activity was observed for EOs obtained at different phenological stages. The IC_50_ values were obtained in the range of 12.14–21.85 µg/mL, in which the highest antioxidant activity was observed for the sample collected at flowering stage while the sample of the fruiting stage showed the lowest antioxidant activity. 

Another method, namely ABTS·^+^, was also employed to estimate the radical scavenging activity of the EOs. The ABTS·^+^ is soluble in water and organic solvents and is applicable in evaluating the antioxidant activity of both lipophilic and hydrophilic compounds [33]. In this method, the significantly (*p* ≤ 0.05) highest and lowest antioxidant activity was recorded for the EOs collected at fruiting (with an EC_50_ value of 134.78 µg/mL) and vegetative (with an EC_50_ value of 142.33 µg/mL) stages, respectively.

In the reducing power assay, the highest reducing power was observed for the EOs collected at the flowering stage (with an EC_50_ value of 36.74 µg/mL) while the lowest reducing power was recorded for the sample obtained at fruiting (with an EC_50_ value of 48.79 µg/mL). In this method, the ferric ion is reduced to the ferrous form by the components of EO on the basis of their respective reducing power. Previous reports have confirmed that the reducing power of EOs might be powerfully linked with their antioxidant activity [64,65]. In the ABTS assay we observed different results with respect to the DDPH assay, with the best activity being displayed by the EO samples obtained at fruiting and flowering (Table 3). This difference in the antioxidant activity can be related to different mechanisms of the antioxidant reactions in the assays, the stereoselectivity of the radicals and differential solubility of EO constituents [36]. Notably, the EO obtained at fruiting contained the highest amount of the highly hydrophobic *p*-cymene (14.7%) (Table 3) that may contribute to boosting the activity of this sample due to its documented efficacy as an antioxidant agent [63].

Our results are in agreement with the findings of Boukhris et al. [66] who investigated the antioxidant activity of *Pelargonium graveolens* L. EOs at different phenological stages, demonstrating a higher antioxidant activity for the sample obtained at the flowering stage. Furthremore, Salem et al. [67] showed the highest antioxidant activity of *Eucalyptus globulus* Labill. EOs at the flowering stage. The antioxidant activity of *S. macrantha* EOs is linked to the presence of the phenolic monoterpenes thymol and carvacrol and their precursor *p*-cymene which are able to scavenge different radical [36,53,68]. The highest antioxidant activity of the EO from the flowering stage is related to the concomitant presence of thymol and carvacrol which can act synergistically as radical scavengers [69] due to the presence of hydroxyl groups in their structures [70]. Oxidative degradation can occur in food matrices during storage; notably the formation of free radicals can induce lipid peroxidation of important constituents of the food matrix such as unsaturated fatty acids [71]. It has been reported that oxidative deterioration is reduced in meat when pre-treated with oregano and sage EOs. Thus, the importance of the use of certain EOs as food preservatives is related to their scavenging properties towards the reactive oxygen species (ROS) while increasing the food shelf life [72,73].

## 4. Conclusions

EOs of *S. macrantha* collected at three phenological stages showed a variation in chemical compositions and biological activities. The strongest antibacterial activity against four important foodborne bacterial strains was obtained for the EO collected at the flowering stage. Alike, this EO exerted the best antioxidant activity according to the DPPH and reducing power assays. The higher biological activities explained by this EO was justified by the presence of higher amounts of the phenolic monoterpenes carvacrol and thymol. On this basis, plants of *S. macrantha* should be preferentially collected during flowering to assure their potentiality to be used on an industrial level as a source of preservative agents. Future encapsulation studies together with a toxicological assessment of this EO should be carried before any application on an industrial level.

## Figures and Tables

**Table 1:** The chemical compositions of the essential oils of *Satureja macrantha* at different growth stages.

No.	Compound	RI ^a^	RI ^b^	Area (%) ^c^		
				Vegetative	Flowering	Fruiting
1	α-Thujene	928	929	0.6	0.3	- ^d^
2	α-Pinene	935	935	0.8	0.4	0.4
3	Camphene	938	937	0.6	0.4	0.5
4	3-Octanone	959	960	0.5	0.3	0.3
5	Sabinene	969	968	0.5	-	0.3
6	β-Pinene	980	979	0.3	0.2	0.1
7	Myrcene	994	992	0.7	0.5	0.5
8	α-Phellandrene	1002	1003	-	1.0	0.8
9	δ-3-Carene	1009	1011	-	0.2	0.1
10	α-Terpinene	1015	1017	1.2	0.9	0.9
11	*p*-Cymene	1021	1021	10.1	11.1	14.7
12	(*E*)-β-Ocimene	1025	1026	1.1	0.3	0.3
13	γ-Terpinene	1054	1058	9.1	7.9	8.0
14	α-Terpinolene	1188	1189	0.2	0.1	0.2
15	Linalool	1094	1095	1.0	-	0.6
16	cis-β-Terpineol	1139	1140	-	-	0.1
17	Camphor	1140	1141	-	-	0.2
18	Borneol	1160	1166	4.9	-	3.8
19	*cis*-3-Cyclohexen-1-ol, 4-methyl-1-(1-methylethyl)	1195	1195	0.6	0.6	0.6
20	Methyl chavicol	1196	1196	-	-	0.4
21	Carvacrol methyl ether	1240	1244	3.1	2.9	2.8
22	(*E*)-Cinnamaldehyde	1260	1266	-	-	0.2
23	Thymol	1290	1291	0.2	16.5	2.0
24	Carvacrol	1295	1297	48.2	42.7	45.6
25	Isoledene	1372	1373	-	-	0.1
26	α-Copaene	1375	1374	-	0.3	0.2
27	β-Bourbonene	1387	1388	0.2	0.3	0.2
28	Carvacrol acetate	1390	1391	0.8	0.3	-
29	α-Cubebene	1340	1346	0.2	-	-
30	(*E*)-Caryophyllene	1418	1420	1.9	2.3	1.2
31	Aromadendrene	1438	1440	0.9	1.3	1.2
32	α-humulene	1450	1452	-	0.2	0.2
33	α-Amorphene	1478	1480	1.0	1.1	-
34	Germacrene D	1483	1485	1.5	2.0	1.1
35	7-epi-α-Selinene	1496	1495	-	-	0.2
36	Bicyclogermacrene	1497	1499	3.3	2.9	1.5
37	β-Bisabolene	1500	1504	0.4	0.8	0.3
38	(*E*,*E*)-α-Farnesene	1506	1507	-	-	0.1
39	*cis*-α-Bisabolene	1508	1511	-	0.2	0.1
40	α-Calacorene	1540	1542	-	-	0.1
41	Spathulenol	1579	1577	2.1	-	1.9
42	Caryophyllene oxide	1580	1581	0.9	1.0	1.1
Total identified (%)				96.7	99.1	92.5
Yield (%, *w*/*w*)				1.5	1.8	1.4
Grouped compounds (%)						
Monoterpene hydrocarbons				25.0	23.4	27.0
Oxygenated monoterpenes				58.7	63.0	55.6
Sesquiterpene hydrocarbons				9.4	11.4	6.2
Oxygenated sesquiterpenes				3.0	1.0	3.0
Others				0.5	0.3	0.9

^a^ Retention index calculated relative to a series of n-alkanes (C8-C20) on capillary HP-5MS column. ^b^ Literature retention indices taken from Adams (2007). ^c^ Percentage of the identified compounds. ^d^ not detected.

**Table 2:** Antibacterial properties of the essential oils of *Satureja macrantha* at different growth stages.

Bacteria Strains		Vegetative Stage		Flowering Stage		Fruiting Stage	
		MIC ^a^	MBC ^b^	MIC	MBC	MIC	MBC
Gram-positive	*Staphylococcus aureus*	10	10	5	7.5	10	10
	*Enterococcus faecalis*	60	80	20	20	40	40
Gram-negative	*Enterobacter aerogenes*	40	40	7.5	10	20	20
	*Escherichia coli*	20	20	10	10	20	20

^a^ MIC: minimum inhibitory concentration (µg/mL). ^b^ MBC: minimum bactericidal concentration (µg/mL).

**Table 3:** Antioxidant activity of essential oils of *Satureja macrantha* at different phenological stages.

Phenological Stage	DPPH (IC_50_, µg/mL)	ABTS (EC_50_, µg/mL)	Reducing Power (EC_50_, µg/mL)
Vegetative	20.69 ± 1.05 b	142.33 ± 6.47 a	42.55 ± 1.84 c
Flowering	12.14 ± 0.63 c	135.25 ± 5.63 b	36.74 ± 1.56 d
Fruiting	21.85 ± 1.02 b	134.78 ± 4.83 b	48.79 ± 2.47 a
Positive control (BHT)	25.76 ± 1.98 a	110.17 ± 4.12 c	40.32 ± 1.69 b

Data are represented as mean ± standard deviation (SD). Different letters in a column indicate significant (*p* ≤ 0.05) differences between the means.
